# Genetic Resistance to *Bacillus thuringiensis* Alters Feeding Behaviour in the Cabbage Looper, *Trichoplusia ni*

**DOI:** 10.1371/journal.pone.0085709

**Published:** 2014-01-21

**Authors:** Ikkei Shikano, Jenny S. Cory

**Affiliations:** Department of Biological Sciences, Simon Fraser University, Burnaby, British Columbia, Canada; University of Tennessee, United States of America

## Abstract

Evolved resistance to xenobiotics and parasites is often associated with fitness costs when the selection pressure is absent. Resistance to the widely used microbial insecticide *Bacillus thuringiensis* (*Bt*) has evolved in several insect species through the modification of insect midgut binding sites for *Bt* toxins, and reports of costs associated with *Bt* resistance are common. Studies on the costs of *Bt-*resistance restrict the insect to a single artificial diet or host-plant. However, it is well documented that insects can self-select appropriate proportions of multiple nutritionally unbalanced foods to optimize life-history traits. Therefore, we examined whether *Bt-*resistant and susceptible cabbage loopers *Trichoplusia ni* differed in their nutrient intake and fitness costs when they were allowed to compose their own protein:carbohydrate diet. We found that *Bt-*resistant *T. ni* composed a higher ratio of protein to carbohydrate than susceptible *T. ni. Bt-*resistant males exhibited no fitness cost, while the fitness cost (reduced pupal weight) was present in resistant females. The absence of the fitness cost in resistant males was associated with increased carbohydrate consumption compared to females. We demonstrate a sex difference in a fitness cost and a new behavioural outcome associated with *Bt* resistance.

## Introduction

The evolution of resistance to pathogens, parasites and chemical insecticides is often accompanied by negative pleiotropic effects in the absence of the selection pressure [1]–[3]. Repeated exposure of insect pests to the toxin-forming bacterium *Bacillus thuringiensis* (or its Cry toxins expressed in plants) has resulted in the evolution of resistance [4], [5]. Fitness costs of insect resistance to *Bt* are well-documented and primarily relate to reductions in survival, fecundity, and mass and increases in development time in comparison to unselected susceptible insects [6]–[11].

The most common genetic resistance mechanism is the modification or loss of midgut binding proteins (cadherin or aminopeptidase N) for *Bt* Cry toxins, necessary for the toxins to cause death [12]. Changes to midgut binding proteins are hypothesized to increase midgut membrane permeability to toxic phytochemicals and pathogens (cadherin), and interfere with protein digestion (aminopeptidases) [13]. Thus the magnitude of any cost to *Bt* resistance is likely to be influenced by diet. This has been shown to be the case, with the costs of *Bt* resistance being exacerbated on lower quality or better-defended diets or host plants [14]–[16].

A key tactic in managing the evolution of resistance to *Bt* relies on the use of non-*Bt* refuges [17]–[19]. If the factors that increase the costs of *Bt* resistance and their underlying mechanisms were better understood, this knowledge could potentially be used to enhance resistance management strategies. Most studies of fitness costs associated with *Bt* resistance restrict the insect to a single artificial diet or host-plant. However, in nature, most herbivorous insects have access to foods that vary in nutritional quality, and they can self-compose their diet from multiple, nutritionally unbalanced foods to optimize life-history traits [20], [21]. We hypothesized that midgut-based resistance to *Bt* was likely to alter nutrient intake and that resistant insects may be able to compensate for fitness costs when given a choice of diets. We used the Geometric Framework for nutrition to test our hypotheses by comparing *Bt-*resistant and *Bt*-susceptible lines of cabbage loopers, *Trichoplusia ni* (Hübner). In this approach it is possible to quantify how an organism regulates the intake of two or more food components at the same time using a graphical model [20], [21]. An important component of this approach is that it incorporates the behaviour of the organism, allowing it to choose between diets or whether to stop or continue feeding. *T. ni* has developed resistance to *Bt* as a result of the frequent applications in greenhouses [8], [22]. Resistance to the Cry1Ac toxin has recently been shown to be related to alterations in two midgut aminopeptidase Ns [23], [24]. In addition, *Bt*-resistant *T. ni* have well-documented, context-dependent costs, including reduced survival and pupal weight, and increased development time [8], [15].

## Materials and Methods

### (a) Study Animals

The *Bt-*resistant *T.ni* colony was originally collected from a commercial tomato greenhouse in British Columbia, Canada in 2001 [8], and has since been maintained on a wheat-germ based diet at 25°C and 16∶8 (L:D) photoperiod. The *Bt-*resistant strain used in the present study was collected during the same year and from a greenhouse close to the source of the Cry1Ac resistant strain used to determine alterations in aminopeptidase Ns (labeled T2c in [8]). At the time of collection, both strains were highly resistant to DiPel, a formulation of *Bt* subsp. *kurstaki* containing Cry1Aa, Cry1Ab, Cry1Ac, and Cry2A [8]. The resistant *T. ni* colony used in the present study is routinely selected with 40 KIU ml^−1^ diet *Bt* subsp. *kurstaki* (Dipel 2× DF, Valent Biosciences, Libertyville, IL, USA) every generation to maintain resistance. The *Bt-*susceptible colony is a revertant line obtained by not exposing the resistant line to *Bt* [8]. Inbreeding and genetic drift have been minimized by mass mating high numbers of moths (approximately 200 moths) each generation and periodic back-crossing between the resistant and susceptible colonies. The *Bt-*resistant insects used here were not selected with *Bt* for one generation (Bt*-*RU) to avoid possible transgenerational effects [15]. Bt-RU larvae were 55-fold more resistant to *Bt* than Bt-S at the time of the experiment.

### (b) Artificial Diets

Experimental larvae were reared on the wheat-germ based diet (colony diet) from egg-hatch until exposure to the wheat-germ-free, nutritionally-defined treatment diets. ‘Treatment diets’ consisted of altering the ratios of protein (casein) and digestible carbohydrate (sucrose) that made up 60% of the dry weight in the following proportions (% protein: % carbohydrate): 50∶10, 30∶30, 20∶40, 10∶50. Other ingredients include Wesson’s salt (5%), cholesterol (1.5%), ascorbic acid (1%), sorbic acid (0.5%), sodium alginate (2.5%), sucrose-free Vanderzant vitamin mix (3.5%), wheat-germ oil (1%) and cellulose (25%) (Bio-Serv, Frenchtown, NJ, USA). Diets were suspended in 1.35% agar solution in a 5∶1 agar solution:dry diet ratio. The wheat-germ based ‘colony diet’ also consisted of approximately 60% protein and carbohydrate, and had an approximate protein to digestible carbohydrate ratio of 1 to 1.1.

### (c) Choice Experiments

Freshly moulted final (fifth) instar Bt-S and Bt-RU larvae were weighed and individually provided with two pre-weighed nutritionally suboptimal but complementary diet blocks, and were allowed to self-compose their diet. Three choice experiments were performed to examine the consistency of self-selected macronutrient ratios, with a choice between the highest P:C ratio diet block (50∶10) and one of the carbohydrate-biased or equal ratio diet blocks (i) 50∶10 & 30∶30, ii) 50∶10 & 20∶40, and iii) 50∶10 & 10∶50). A total of 72, 75, and 79 larvae were used for each choice experiment, respectively. The diet was replaced each day until pupation; any diet remaining was collected, dried at 50°C for 24 hrs until constant mass, and then weighed. Daily consumption was estimated by calculating the difference between the dry initial and final remaining mass of the diet blocks. The dry initial mass of the diet blocks was estimated by constructing a regression equation with pre-weighed diet blocks without larvae. All larvae successfully pupated, and the date of pupation was recorded. Three days after pupal initiation, pupae were sexed and then dried at 50°C for 48 hrs until constant mass and then weighed.

### (d) Statistical Analyses

Differences in the cumulative bivariate consumption of protein and carbohydrate by day between Bt-RU and Bt-S were analysed by multiple analysis of covariance (MANCOVA) using Pillai’s trace statistic. Sex and choice experiment were included as fixed effects and initial larval weight was included as a covariate. Univariate analyses of protein and carbohydrate intake were obtained from post hoc analyses as part of the bivariate MANCOVA. Analysis of covariance (ANCOVA) for pupal weight was performed separately. Pupal weight was squared before analysis to normalize the data. Tukey HSD comparison was used to compare means among treatments. Time to pupation was analyzed using accelerated failure-time analysis using a Weibull distribution. For all analyses, all factors and their interactions were fitted initially in the model and non-significant interactions were removed sequentially to produce the final minimal model. SAS 9.3 was used for all analyses (SAS Institute, 2010, Cary, NC, USA). The dataset has been made available as supporting information (Dataset S1).

## Results

**Figure 1:**
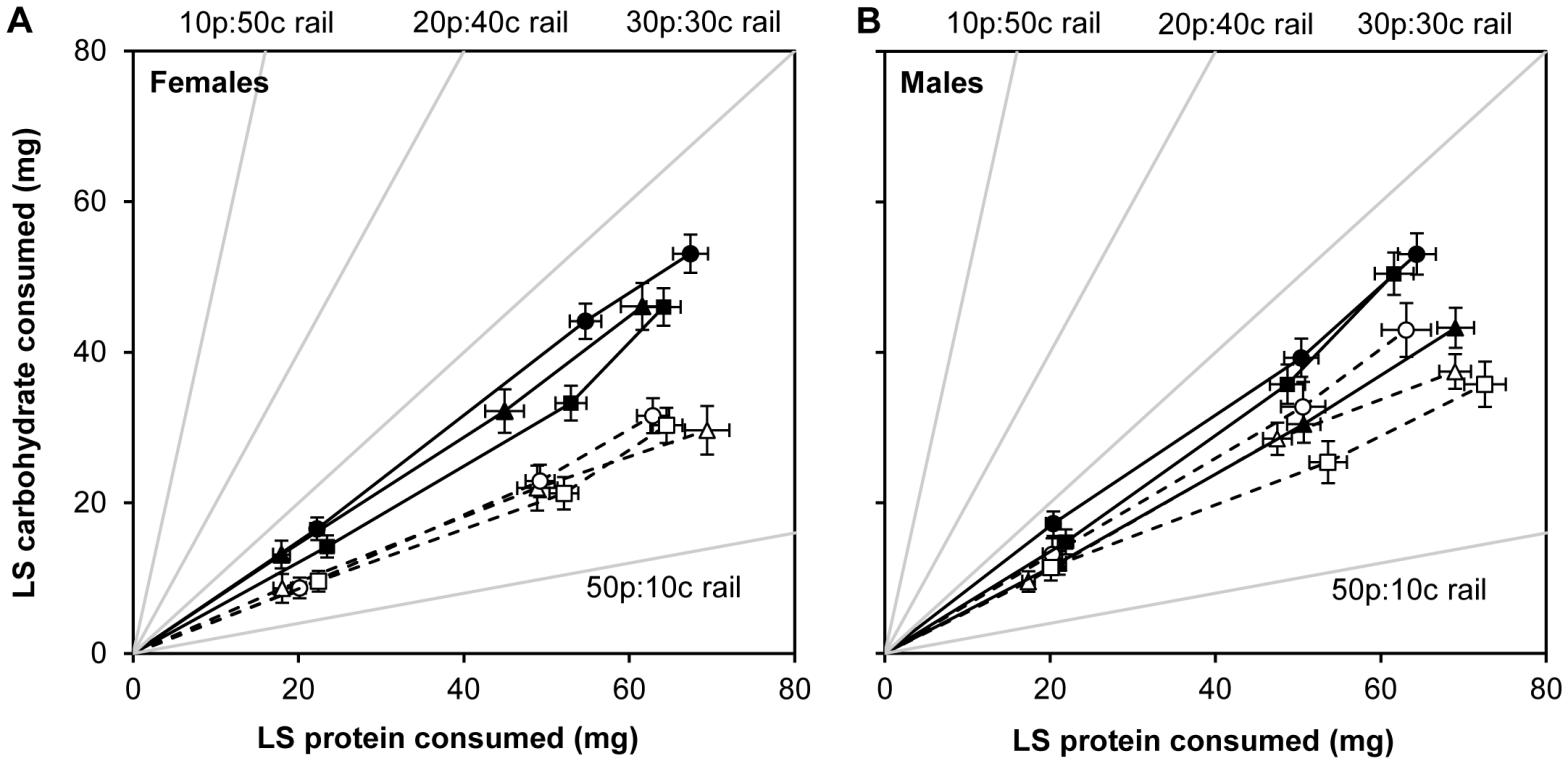
*Bt* resistance alters nutrient intake. Bivariate least squares means (±SE) of protein and carbohydrate intake composed by (A) female and (B) male Bt-S (solid black symbols) and Bt-RU (open symbols). Points along each trajectory correspond to the cumulative intake of protein and carbohydrate over consecutive days. Intake points for day 0–1, days 0–2, and days 0-pupation are shown. The solid gray lines indicate the macronutrient ‘rails’ of each diet block provided. The choices were as follows: 50p:10c & 30p:30c (triangle); 50p:10c & 20p:40c (circle); 50p:10c & 10p:50c (square).

When provided with two nutritionally suboptimal but complementary diet blocks, the intake target of Bt-S larvae was more carbohydrate-rich than Bt-RU larvae (Figure 1; Table 1). Both Bt-S and Bt-RU showed some flexibility in their final intake targets (Days 0-pupation) as there was a significant effect of choice and a marginally significant three-way interaction between colony, choice and sex. It appears males showed more variation in their nutrient intake target than females. The daily variation in feeding between Bt-S and Bt-RU indicates that Bt-S consumed more food (protein and carbohydrate combined) each day than Bt-RU. The mean nutrient intake targets from the three choice experiments for female Bt-S and Bt-RU were 64.4p:48.4c and 65.6p:30.5c respectively, and 65.0p:48.9c and 68.2p:38.7c respectively for male Bt-S and Bt-RU. Thus, female Bt-RU consumed a higher ratio of protein to carbohydrate than male Bt-RU (2.1∶1 and 1.8∶1 respectively), while both sexes of Bt-S consumed the same P:C ratio (1.3∶1).

**Table 1:** MANCOVA analyses for protein and carbohydrate intake by Bt-S and Bt-RU from the three choice experiments.

	Protein and carbohydrate intake								
	Day 0–1			Days 0–2			Days 0-pupation		
source	d.f. (Num, Den)	*F-*value	*P-*value	d.f. (Num, Den)	*F-*value	*P-*value	d.f. (Num, Den)	*F-*value	*P-*value
initial weight	2, 219	0.45	0.64	2, 218	10.90	<0.0001	2, 218	0.40	0.67
sex	2, 219	5.54	<0.01	2, 218	2.06	0.13	2, 218	5.84	<0.01
colony	2, 219	41.33	<0.0001	2, 218	26.82	<0.0001	2, 218	40.73	<0.0001
choice expt	4, 440	2.39	0.05	4, 438	5.93	<0.0001	4, 438	2.68	0.03
colony×choice expt	4, 436	0.67	0.62	4, 434	2.16	0.07	4, 434	2.05	0.09
colony×sex	2, 216	0.75	0.47	2, 218	5.93	<0.01	2, 218	4.88	<0.01
choice expt×sex	4, 430	1.70	0.15	4, 430	0.51	0.73	4, 430	0.69	0.60
colony×choice expt×sex	4, 426	1.43	0.22	4, 426	2.39	0.05	4, 426	2.36	0.05

Analyses were performed on each of the three cumulative intake points (days 0–1, 0–2, and 0-pupation). Non-significant interactions (p≥0.05) were removed sequentially.

colony = susceptible vs. resistant.

**Figure 2:**
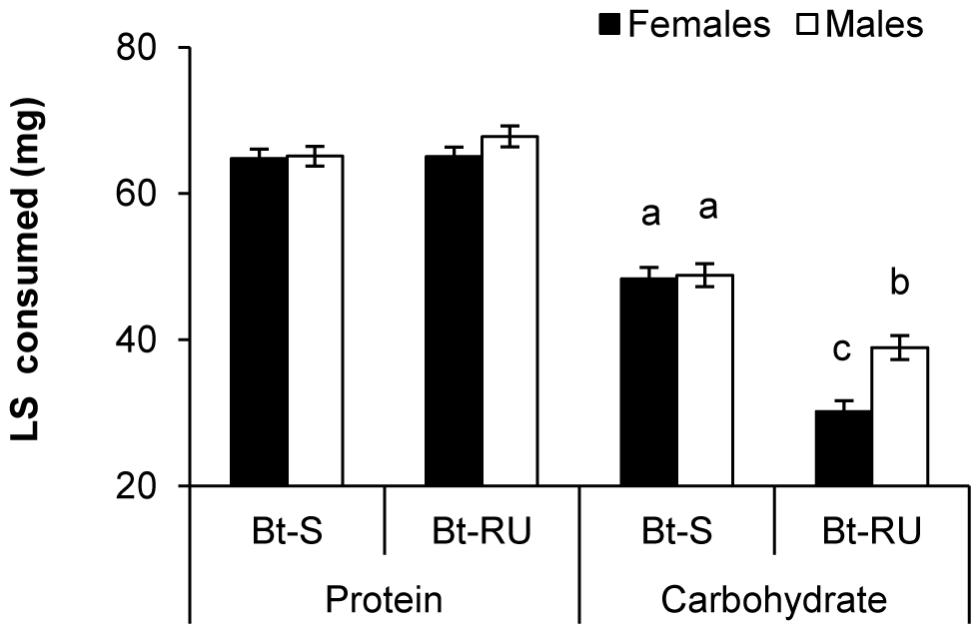
Sex differences in compensatory feeding. Least squares means (±SE) of total protein and carbohydrate consumption for female and male Bt-S and Bt-RU. Different letters indicate significant differences from Tukey HSD comparison (p<0.05).

This prompted an examination of the univariate ANCOVA results for protein and carbohydrate intake separately. Protein consumption showed a significant three-way interaction that is likely due to differences in the direction of the effects among choice experiments. However, there were no significant differences when compared by Tukey HSD (Figure S1). There were no two-way interactions or main effects in the model either. In contrast, there was no three-way interaction for carbohydrate intake, but a strong effect of colony, sex and their interaction (Table 2). Bt-S larvae consumed more carbohydrate than Bt-RU larvae; however, whereas both Bt-S sexes consumed the same, Bt-RU males consumed more carbohydrate than Bt-RU females (Figure 2). Therefore, the higher P:C ratio composed by Bt-RU resulted from similar protein intake as Bt-S, but reduced carbohydrate intake, more so for females than males.

**Table 2:** ANCOVA results for protein and carbohydrate intake, and pupal weight. Non-significant interactions (p≥0.05) were removed sequentially.

source	protein			carbohydrate			pupal weight squared		
	d.f.	*F-*value	*P-*value	d.f.	*F-*value	*P-*value	d.f.	*F-*value	*P-*value
initial weight	1, 213	0.85	0.36	1, 219	1.6	0.20	1, 219	16.94	<0.0001
sex	1, 213	2.74	0.10	1, 219	6.28	0.01	1, 219	9.59	<0.01
colony	1, 213	1.62	0.20	1, 219	90.23	<0.0001	1, 219	40.77	<0.0001
choice expt	2, 213	1.27	0.28	2, 219	4.25	0.02	2, 219	10.47	<0.0001
colony×choice expt	2, 213	2.70	0.07	2, 217	1.07	0.35	2, 217	2.79	0.06
colony×sex	1, 213	1.45	0.23	1, 219	7.05	<0.01	1, 219	14.11	<0.001
choice expt×sex	2, 213	1.22	0.30	2, 215	0.30	0.74	2, 215	0.60	0.55
colony×choice expt×sex	2, 213	4.09	0.02	2, 213	1.12	0.33	2, 213	1.79	0.17

colony = susceptible vs. resistant.

**Figure 3:**
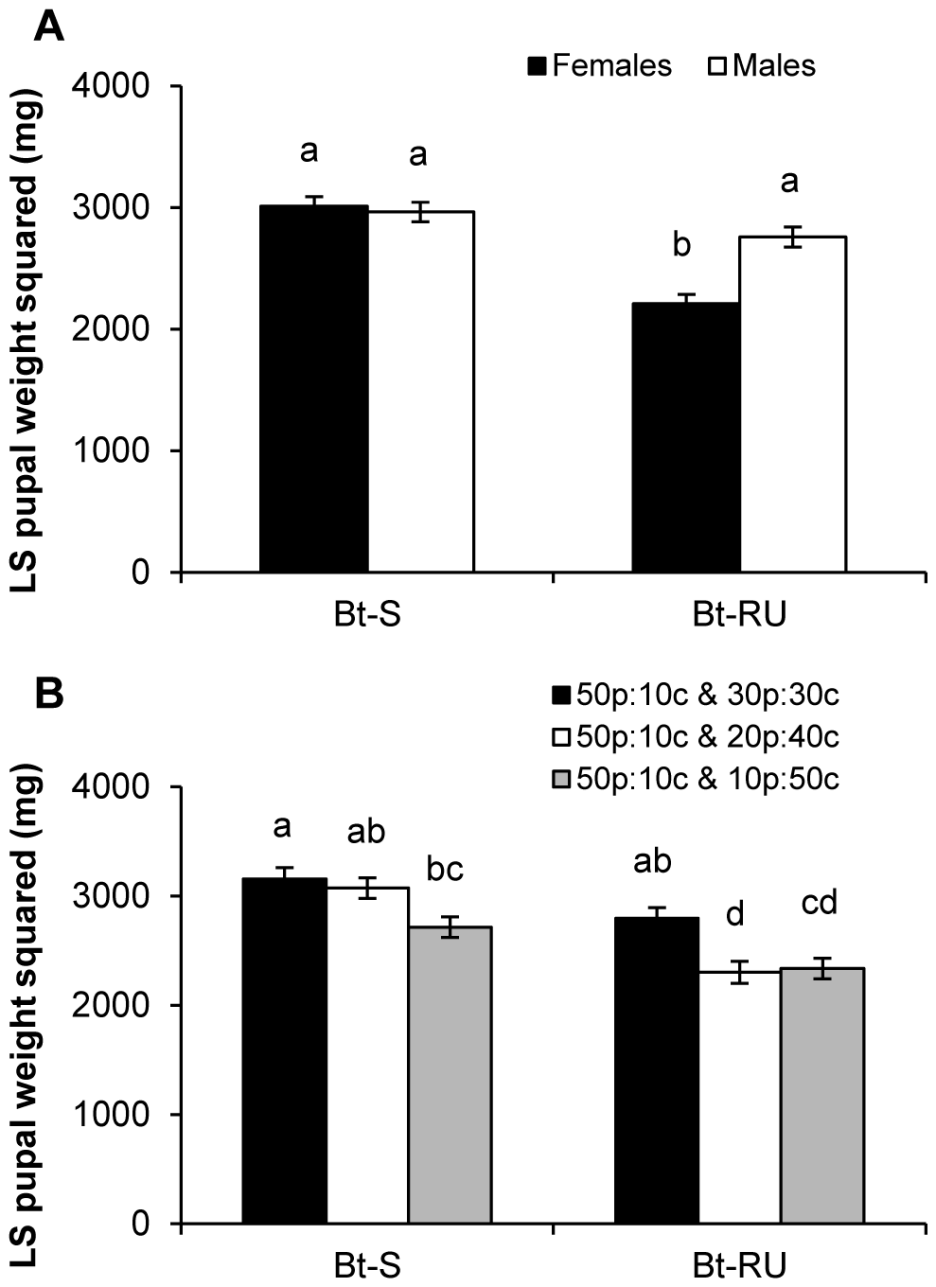
Potential for compensatory feeding that reduces fitness cost. (A) least squares means (±SE) of square-transformed dry pupal weight for female and male Bt-S and Bt-RU. (B) Least squares means (±SE) of square-transformed dry pupal weight for Bt-S and Bt-RU for each choice experiment. Different letters indicate significant differences from Tukey HSD comparison (p<0.05).

**Figure 4:**
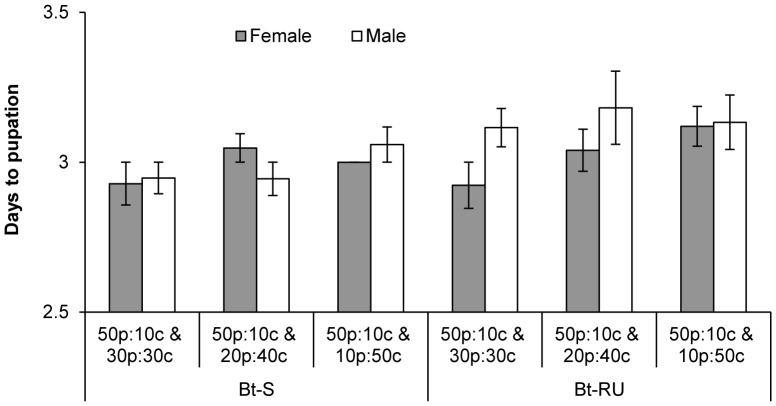
Delayed development accompanies compensatory feeding. Mean number of days (±SE) to initiate pupation in the final instar by Bt-S and Bt-RU.

Higher carbohydrate consumption by Bt-RU males was associated with heavier pupal mass, such that they weighed the same as Bt-S pupae (colony by sex interaction, Figure 3A, Table 2). Pupal weight of Bt-S and Bt-RU were also significantly affected by choice experiment such that pupal weights decreased as the distance in nutritional space between the protein-biased diet (50p:10c) and the carbohydrate-biased diet (30p:30c, 20p:40c and 10p:50c) became greater. We examined this effect further by performing a Tukey HSD comparison on the marginally significant interaction between choice experiment and colony. The analysis revealed that Bt-S maintained heavy pupal weights on the two choice experiments that were closer together in nutritional space (50p:10c & 30p:30c and 50p:10c & 20p:40c), while Bt-RU had high pupal weight only when the nutritional space between the two choices was closest (50p:10c & 30p:30c; Figure 3B). Differences in pupal weight could be explained by a significant three-way interaction for the number of days taken to initiate pupation (Table 3). Male Bt-RU achieved the same pupal weight as Bt-S, but consistently fed for a longer period of time to reach pupation (Figure 4). On the other hand, female Bt-RU did not take longer to develop except on the most skewed diet choice (50p:10c & 10p:50c).

**Table 3:** Accelerated failure-time analysis for days to pupation.

source	days to pupation		
	d.f.	*X^2^*	*P-*value
initial weight	1	0.01	0.90
sex	1	4.57	0.03
colony	1	22.10	<0.0001
choice expt	2	9.08	0.01
colony×choice expt	2	0.70	0.70
colony×sex	1	3.99	0.05
choice expt×sex	2	2.89	0.24
colony×choice expt×sex	2	8.14	0.02

## Discussion

Allowing susceptible and resistant *T. ni* larvae to self-compose their diets in the final instar revealed that while both Bt-S and Bt-RU larvae regulate protein and carbohydrate intake, they differed in their nutrient intake target ratio. Bt-RU larvae ate less carbohydrate than Bt-S but similar quantities of protein. Bt-S composed a more balanced diet while Bt-RU larvae composed a P:C ratio that was considerably higher in protein (1.3p:1c and 2p:1c respectively). Broadly, this is in line with other Lepidoptera, in that protein intake is either equal to or greater than carbohydrate ingestion [20]. The reduced carbohydrate intake was primarily accounted for by resistant females eating less carbohydrate than the males. Reduced food intake by Bt-RU that resulted from reduced carbohydrate intake suggests that the two colonies may be adopting different lifestyles, such that one feeds more and grows faster while the other feeds more conservatively and grows slower. Such differences in lifestyles are often associated with different costs. For example, higher foraging rates can be associated with higher predation rates [25].

Bt-RU females weighed less than the susceptible pupae of both sexes, whereas resistant males weighed the same. However, male Bt-RU achieved a larger pupal weight at the cost of longer development time. Male pupal weight is weakly heritable and positively correlated with *Bt* susceptibility in *Bt-*resistant *T. ni* [26]. *T. ni* males mate multiple times and it is hypothesized that increased weight improves mating success and frequency [26]. Female pupal weight is generally correlated with fecundity; however, no relationship with *Bt* susceptibility has been found in *T. ni* [26]. The reduction in female pupal weight found here is thus harder to explain but does suggest an inability to compensate for fecundity loss. One hypothesis is that since feeding on a diet with a higher P:C ratio increases immune activity and survival after pathogen challenge in another Lepidopteran species [27], selection pressure may be higher for female Bt-RU to survive *Bt-*challenge, and thus ingest more protein, whereas males are selected for mating success and frequency. Virally-infected caterpillars have shown similar behaviour to improve their likelihood of surviving infection by reducing carbohydrate consumption while maintaining protein consumption [28]. Achieving higher immune activity through higher P:C consumption may also allow female Bt-RU to have enhanced immunity in the next generation, as induced *Bt* tolerance has been shown to be transferred to the next generation by maternal effects [29].

Given that Bt-RU and Bt-S are back-crossed to maintain genetic similarity and have been continuously reared on the same diet, these differences in feeding behaviour are consistent with an association with resistance to *Bt*. Studies with more lines of Bt-resistant *T.ni* and other species are needed to confirm the generality of this finding. Investigation of precise mechanisms are beyond the scope of this paper but altered nutrient intake might result from changes in the feedback mechanism that provides information about the nutrient content of food and chemical composition of the haemolymph [30], or from other, as yet identified, mechanisms resulting from the modification of midgut binding proteins [22], [23]. It is important to mention that the use of casein as the only protein source (from an animal) and sucrose as the only carbohydrate source are not representative of the natural dietary nutrients for *T. ni*. Whether the results from this study hold in situations where other protein and carbohydrate sources are available or on plants differing in nutritional quality warrant further investigation.

Herbivorous insects have access to a variety of plants and plant parts that vary in nutritional content. Our experiments demonstrate that *T. ni* larvae will self-compose a specific balance of nutrients, and that the evolution of pathogen resistance can change feeding behaviour to alter the nutrient intake target. Consumption of a higher ratio of protein by Bt-RU suggests that they may seek out plant parts higher in protein, adding to their destructive potential as pests by preferentially consuming the reproductive and younger parts of plants that are protein rich. Alternatively, such behaviour might enhance refuge-based resistance management strategies if these plant parts contain higher levels of defensive phytochemicals. Understanding the behavioural and physiological outcomes of resistance selection is important in predicting the destructive potential of resistant insects and the success of resistance management strategies.

## Supporting Information

Figure S1 **Variability of protein intake.** Least squares means of the three-way interaction between colony, sex, and choice experiment on Table 2. Same letters indicate no significant differences between means determined by Tukey HSD analysis (p>0.05).
(DOCX)

Dataset S1 **Data of nutrient intake and development measures.**
(XLS)
