# Supplementary material for: Genetic Resistance to Bacillus thuringiensis Alters Feeding Behaviour in the Cabbage Looper, Trichoplusia ni
Source: PLoS One. 2014 Jan 21;9(1):e85709. doi: 10.1371/journal.pone.0085709 (PMC3897465; doi:10.1371/journal.pone.0085709)
Supplement: Supplementary file 1 [file pone.0085709.s001.docx]

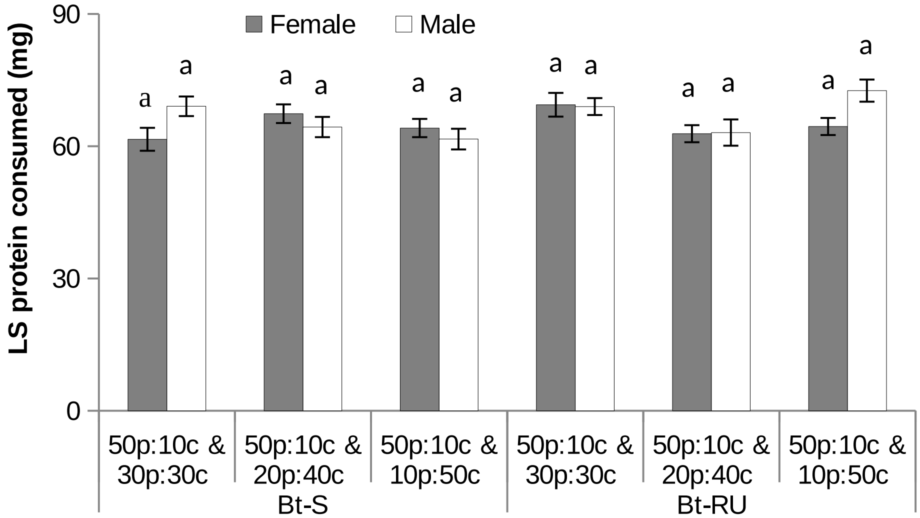

LS protein consumed (mg)
90
60
30
0
Female
Male
a
a
a
a
a
a
a
a
a
a
a
a
50p:10c & 30p:30c
50p:10c & 20p:40c
50p:10c & 10p:50c
Bt-S
50p:10c & 30p:30c
50p:10c & 20p:40c
50p:10c & 10p:50c
Bt-RU
